# Regional Variations of Fertility Control Behavior among Rural Reproductive Women in Bangladesh: A Hierarchical Analysis

**DOI:** 10.3390/bs8080068

**Published:** 2018-07-31

**Authors:** Muhammed Ashraful Alam, Kanittha Chamroonsawasdi, Natkamol Chansatitporn, Chokchai Munsawaengsub, Md Shafiqul Islam

**Affiliations:** 1 Doctoral Candidate in Doctor of Public Health Program, Department of Family Health, Faculty of Public Health, Mahidol University, Bangkok 10400, Thailand; dralam1874@gmail.com; 2 Department of Family Health, Faculty of Public Health, Mahidol University, Bangkok 10400, Thailand; chokchai.mun@mahidol.ac.th; 3 Department of Biostatistics, Faculty of Public Health, Mahidol University, Bangkok 10400, Thailand; nutkamol.cha@mahidol.ac.th; 4 National Institute of Preventive and Social Medicine (NIPSOM), Dhaka 1212, Bangladesh; shafiqbd2010@gmail.com

**Keywords:** regional variation, fertility control, hierarchical analysis, Bangladesh

## Abstract

Women’s fertility decision is quite difficult in male-dominant rural culture due to their poor reproductive autonomy. A cross-sectional survey was conducted in rural community of Bangladesh between November 2017 and February 2018 among 1285 respondents selected by multi-stage stratified sampling to explore regional variations of rural women’s fertility control behavior and its determinants using hierarchical and other inferential statistics. Data collection was done by face-to-face interview using questionnaire. Average parity was 2.5 per woman and 41% respondents had three or more children. About 60% of them used modern contraceptives (MCs) and oral contraceptive pill (OCP) was their first choice. Male participation in contraceptive use was less than 5%. Regional variation, women’s empowerment, fertility control knowledge, family planning (FP) attitude, social influence, perceived behavioral control (PBC) and fertility intention were significant predictors of fertility control behavior (*p* < 0.05). Significant regional variations were determined in fertility control behavior of rural women (*p* < 0.05). Almost all of its predictors explained by Theory of Planned Behavior (TPB) also showed significant regional variations (*p* < 0.05). Current fertility control policy should be strengthened more not only to improve fertility behavior of rural women but also to establish regional equity in fertility control by improving their reproductive decision-making in a rational way.

## 1. Introduction

Fertility control behavior is the planned outcome of fertility decision that helps clients to choose an appropriate contraceptive method in a rational way according to their expressed needs and situation to carry out their reproductive intentions successfully [1,2]. Use of modern contraceptives (MCs) has been established as the best way to control fertility for long decades [2]. The International Conference on Population and Development (ICPD) in 1994 focused on women’s right on decision-making of fertility control and reproductive autonomy to ensure their equity [2]. Every woman has the right to decide the number, birth spacing and timing of her children, and the means through which she will achieve the desired family size [2,3,4,5,6,7]. Bangladeshi women are far behind in their reproductive decision-making. This problem is more frequently in rural areas and regional variation is an added issue to cope with the vulnerability in fertility decision [8,9]. Fertility control is an urgent issue not only to improve maternal and child health (MCH) but also to ensure country development [10,11].

According to global context, total fertility rate (TFR) in 2015 was 2.5 live births per woman with a huge regional difference [12]. The highest fertility rate was found 4.7 live births per woman in Africa, while the lowest found was 1.6 live births per woman in Europe [12]. Even some Asian countries like Singapore and Japan showed TFR less than 1.5 in 2016 [13]. Contraceptive use has been increasing steadily since 1970 and is currently widespread throughout the world [3]. Globally, modern contraception prevalence rate (CPR) has risen slightly, from 54% in 1990 to 57.4% in 2015 [14,15]. Although contraceptive use has increased in many parts of the world, especially in Asia and Latin America, among least-developed countries like the sub-Saharan region it is still at a low CPR [14,15]. TFR and CPR not only differ among Asian countries but also show regional disparity in different socio-economic groups within countries like Bangladesh, India and Pakistan [8,16,17].

In 2016, Bangladesh had an estimated population of more than 160 million, which makes it the 8th most populous country in the world [13,17]. Population density (people per sq. km) in Bangladesh was last measured at 1236.81 in 2015, according to the World Bank which ranks it 10th in the world [17]. Bangladesh has dramatically improved in fertility control over last few decades. Following a very successful family planning (FP) program, TFR fell from 3.3 in 1999–2000 to 2.3 in 2011 but it remained in the same state of TFR for the last few years [8]. Success was also observed in increased rate of CPR and reduction of unmet needs in Bangladesh but urban–rural gap in fertility indicators is still high [8]. In spite of progress, TFR is much higher (2.4 per woman of reproductive age) and accessibility to healthcare services remains quite poor in remote areas of Bangladesh [8]. Not only urban–rural disparity, persistent gaps in fertility control indicators among different rural areas were still a major concern to policy makers. Bangladeshi rural women are hardly familiar with reproductive autonomy and they are deprived in FP decision-making [8,9]. Previous studies found that inadequate contraceptive knowledge [8], son preference [8,9], male-dominant culture and financial circumstance [8,9] make fertility control behavior problematic for rural women. Though several studies were done previously to identify fertility control behavior and its related factors, the situation in rural Bangladesh was still unclear and needed to be explored more.

Although decision-making in fertility control is a major concern in reproductive autonomy, most of the rural women are not familiar with rational ways of contraceptive decision-making even among the MC users [9]. Fertility control behavior described through modified Theory of Planned Behavior (TPB) can be highly effective to encourage rural women of Bangladesh to take contraceptive decisions in a rational way. TPB is an integrative framework for the prediction of human social behavior. The TPB states that attitudes towards the behavior, perceived norms, and perceived behavioral control (PBC) determine people’s intentions, while people’s intentions predict their behaviors [18,19,20,21]. Actual behavioral control can affect the outcomes of intentions [21]. The psychological determinants of TPB were taken as the predictors of fertility control behavior along with socio-demographic status. Regional variations of fertility control behavior and its predictors described in the study based on TPB were almost untouched in reproductive health research in rural Bangladesh. This study was aimed at identifying regional variations of fertility control behavior among reproductive women in rural Bangladesh with its associated factors applying TPB. Socio-economic status (SES) factors (i.e., age, education, family income and family size), women’s empowerment, fertility preference and contraceptive knowledge were included in this study as background factors of TPB model. FP attitude, PBC, social influence and fertility control intention were taken from core components of TPB. Male dominancy in spousal status was taken as an external control in this study for fertility control behavior of rural women. Regional effect on fertility control behavior was observed through hierarchical model using blocks of SES and core components of TPB. Finally, the regional variations of fertility control behavior and its predictors were observed. With this approach, it is possible to gain a better understanding of women’s rational use of contraceptive to control their fertility. The study will make a platform for policy makers for prompt actions to reduce regional disparity in fertility control as well as to improve fertility control behavior of women in rural Bangladesh in the near future.

## 2. Materials and Methods

### 2.1. Study Design

This cross-sectional survey was conducted in rural areas of three divisions (i.e., Dhaka, Rajshahi and Chittagong) of Bangladesh.

### 2.2. Sample Size

The study was conducted among 1285 married women of reproductive age (15–49 years) having at least one child currently living together with their husbands in the rural area at least for 12 months before the survey.

### 2.3. Sampling Method

**Figure 1:**
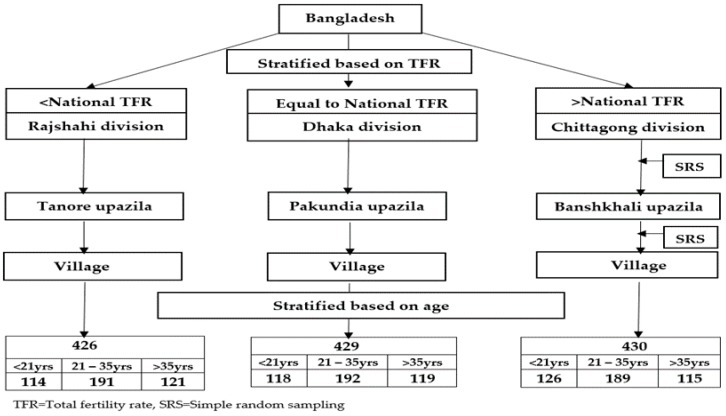
Multistage sampling.

The respondents were selected by using multistage sampling technique (Figure 1). Initially, one division was selected from each stratum by stratified sampling on the basis of national TFR. The process helped to explore regional variation. Then one upazila was randomly selected from each stratum. After selection of one upazila, village was selected from each upazila again by simple random sampling (SRS). After selection of village, age distribution of the respondents was under consideration to make it representative for all rural women of reproductive age. Three age groups were considered: equal or less than 20 years, 21–35 years and more than 35 years. Respondents were randomly selected in a non-proportionate way from each age group according to the sampling frame considering age distribution in the rural community. Around 430 respondents were randomly selected from the village or villages of each division based on the inclusion criteria using FP registration records from the local FP office.

### 2.4. Data Collection

Data were collected by the researcher and 4 trained interviewers with previous exposure in health research between November 2017 and February 2018. Face-to-face interview was done through a structured questionnaire having 10 parts. Data collectors asked questions orally and filled out the responses. Socio-demographic and reproductive characteristics of respondents were described in the first part through 18 items such as age, income, number of children, etc. The second part was used to describe fertility control behavior of rural women using 6 items related to birth control and birth spacing. It was mixed-type, including both Likert as well as dichotomous types of answers. The third part described women’s autonomy using 10 items such as decision-making, economic access and mobility. Women’s fertility preference was assessed in the fourth part using 6 items such as preference for larger family and son preference. Part 5 included 8 items regarding information about contraceptive use and birth spacing to assess fertility control knowledge. Spousal relation was assessed in part 6 through 6 items such as spousal communication and male participation in FP. Part 7 was constructed to assess women’s FP attitude using 8 items. Social influence in fertility control was described by 8 items in part 8. Women’s PBC was assessed in part 9 through 6 items such as self-control on contraceptive-decision for birth control and birth spacing. Intention to fertility control was assessed in part 10 using 6 items such as readiness to use contraceptive and readiness for fertility control. Likert scale was followed from part 3 to part 10, except part 5, where dichotomous type was used. Equal numbers of positive and negative questions were included in the scales of fertility preference, fertility control knowledge, FP attitude and fertility control intention scales and only in PBC scale there were 4 positive and 2 negative questions. Cronbach’s Alpha (α) of fertility control behavior scale was 0.72. Cronbach’s α for women’s empowerment and fertility preference were 0.77 and 0.79, respectively. Cronbach’s α of FP attitude scale was 0.80 and for PBC scale it was 0.87. Cronbach’s α for social influence and fertility control intention were 0.72 and 0.78, respectively. Only spousal status and fertility control knowledge scales showed Cronbach’s α score between 0.6 and 0.7.

### 2.5. Data Analysis

Data processing and analyses were done using Statistical Package for Social Sciences (SPSS) version 20. Frequencies, percentage, mean and standard deviation (SD) were used for descriptive statistics. Hierarchical regression model was used to describe significance of predictors as well as regional effect on fertility control behavior. Kruskal–Wallis H and one-way analysis of variance (ANOVA) tests were used to describe the regional variations of fertility control behavior of rural women with its predictors. Factors related to fertility control behavior of rural women were arranged in groups for hierarchical analysis (Table 1).

### 2.6. Ethical Consideration

Ethical clearances were taken from the Ethical Committee of Mahidol University (COA. No. MUPH 2017-193) and also from the Bangladesh Medical Research Council (BMRC) (Reg. No. 07427092017) to conduct the study.

## 3. Results

The typical rural picture was found to describe the socioeconomic status of the respondents. Nearly half of the respondents (44.5%) were in the group of 21–35 years. The average age of the respondents was 27.7 years with SD of 7.8 years (Table 2). Around half of the husbands (52.2%) were in the group of 26–35 years and their mean age was 34.8 years with SD of 8.7 years (Table 2). The majority (90%) of the respondents were Muslims and the rest of them were Hindus. About 22% of the respondents were illiterate or without academic learning and nearly half of them (48.9%) were literate up to primary level. Men were more illiterate than their women counterpart. The mean of monthly family income was 10,480 Taka with SD of 5261 Taka (Table 2). The majority (90%) of the respondents were housewives. Among the husbands, more than half of them (55.2%) were agricultural workers and 17.4% were day laborers. Almost two-thirds of respondents (62.8%) lived in extended family. The average family size was 5.5 with ± 1.6 SD (Table 2).

Among the respondents, about 80% had a history of early marriage and mean age of marriage was 16.6 years with SD of 1.1 years. Among the respondents, 78.6% had adolescent pregnancy and mean age of first pregnancy was 18.2 years with SD of 1.3 years. Around 43% had a history of pregnancy more than two times and 7% respondents had a history of abortion. Around 42% had a history of delivery more than two times and average parity was 2.5 per woman. Women having more than two living children were 41% and among the respondents, almost half of them (48.1%) conceived within two years of marriage. Average initial gap of first pregnancy from their marital age was 1.6 years with SD of 0.7 year (Table 3).

Almost half of them (48.6%) regularly used MCs and 11.3% irregularly used. Among the respondents, around 27% shared about switching in contraceptive use. Only around 8% used natural methods. Among the users of MCs, more than half (54.2%) used oral contraceptive pills (OCPs), 22.5% used injections, 3.6% used implant and 2.7% used Copper-T. Among the users of MCs, condom was used only in 7.5% cases and 9.5% gave a history of using permanent methods of contraceptives, either tubal ligation or vasectomy (Table 3). 

The three-stage hierarchical multiple regression revealed that background factors at stage one contributed significantly to the regression model (F (8, 1276) = 199.49, *p* < 0.001) and accounted for 55.6% of the variation in fertility control behavior of rural women (Table 4). Next, core items according to TPB like FP attitude, social influence, PBC, spousal status and fertility intention were entered into the second model. Introducing these variables explained an additional 10.8% of variation in fertility control behavior and this change in R² was significant (F (5, 1271) = 81.98, *p* < 0.001). Finally, the area was added to find out the significant effect of regional variation on fertility control behavior. The inclusion of area to the regression model explained an additional 0.2% of the variation in fertility control behavior. Although this change in R² was small, it was significant (F (2, 1269) = 4.06, *p* < 0.05).

Women’s age, family income, empowerment and fertility preference showed significance predicting fertility control behavior in the first model of background issues. Age and women’s empowerment persisted as significant predictors in the second model. Fertility control knowledge from background issues also showed significance in the second model. FP attitude, social influence, PBC and fertility intention were highly significant to predict fertility control behavior among the core items of TPB in the second model. When all independent variables were included in stage three of the regression model, socio-economic variables showed no significance predicting fertility control behavior of rural women. Only women’s empowerment and fertility control knowledge from background issues were significant to predict fertility control behavior in the final stage of the regression model (*p* < 0.001) (Table 4). FP attitude, social influence, PBC and fertility control intention were highly significant to predict fertility control behavior in the final stage of the regression model (*p* < 0.001) (Table 4). Spousal status showed no significance predicting fertility control behavior. Finally, the regional variation was determined as an important factor showing significance to predict fertility control behavior of rural women. Fertility control behavior of respondents of Dhaka and Rajshahi divisions were significantly different (*p* < 0.05) from fertility control behavior of rural women of Chittagong division. 

The average score of women’s fertility control behavior for all divisions was 8.9 (±4.6). The average women’s empowerment score and average fertility preference score at the national level were 34.3 (±5.2) and 20.1 (±3.5), respectively. Mean score of fertility control knowledge was 5.6 (±1.7) and average FP attitude score among total respondents was 30.1 (±3.3). The average social influence score and average PBC score at national level were 29.0 (±3.6) and 21.6 (±4.3), respectively. The average fertility control intention score was 20.9 (±3.6) and mean score of spousal status among total respondents was 7.6 (±3.1) (Table 5).

Kruskal–Wallis H test was conducted to determine regional variations of women’s fertility control behavior along with its few predictors such as women’s empowerment, spousal status, perceived behavioral control and fertility control intention. In the case of regional variation of fertility control behavior of rural women, Kruskal–Wallis test showed that there was a significant difference of means, χ^2^ (2) = 49.41, *p* < 0.001, with a mean rank fertility control behavior score of 544.28 for Chittagong, 669.55 for Dhaka and 715.91 for Rajshahi. Chittagong was very highly significantly different from Dhaka (*p* < 0.001) and Rajshahi (*p* < 0.001) but Dhaka and Rajshahi were not significantly different (*p* > 0.05). Significant regional variations of women’s empowerment (χ^2^ (2) = 64.03, *p* < 0.001), spousal status (χ^2^ (2) = 26.72, *p* < 0.001), PBC (χ^2^ (2) = 17.42, *p* < 0.001) and fertility control intention (χ^2^ (2) = 18.96, *p* < 0.001) were also confirmed by Kruskal–Wallis H tests. Post hoc tests confirmed the vulnerability of Chittagong from Rajshahi and Dhaka (Table 5).

Significant regional variations of fertility preference (F (2, 1282) = 6.09, *p* < 0.01), FP attitude (F (2, 1282) = 12.58, *p* < 0.001) and social influence (F (2, 1282) = 25.10, *p* < 0.001) in fertility control were confirmed through one-way ANOVA tests. Tukey post hoc tests revealed the vulnerability of Chittagong from other divisions in fertility preference, FP attitude and social influence. In the case of fertility control knowledge, there was no statistically significant difference found among rural women of three divisions as determined by one-way ANOVA (F (2, 1282) = 2.12, *p* > 0.05) with a mean fertility knowledge score of 5.5 for Chittagong, 5.6 for Dhaka and 5.8 for Rajshahi. Although there was no significant difference, Rajshahi showed higher score (Table 5).

## 4. Discussion

This study showed that addressing women’s fertility decision remained challenging in a male-dominant rural community. Fertility control behavior as well as its predictors were explained through TPB in different backgrounds. Less than two-thirds (59.9%) of the respondents were MC users. The rate of MC use was still slightly higher in the study area in comparison to a recent Bangladesh Demographic and Health Survey (BDHS) [8]. One of the main reasons might be the inclusion of rural women having at least one child. Almost two-thirds (65.5%) of the respondents of Rajshahi used MC and 59.5% of the respondents of Dhaka were MC users. Chittagong showed the lowest percentage (54.9%) of MC users. Although the contraceptive prevalence was not very high in this study, the regional variation of contraceptive use and parity of this study is quite similar to the findings of recent BDHS [8] and other FP papers [22,23]. Alpu and Fidan also identified region and place of residence as significant factors in fertility control with education and women’s attitude in their study on contraceptive use among married women in Turkey [24]. Thang and Huong also described regional variations of contraceptive use in Vietnam [25].

OCP was the most common method in contraceptive use, followed by injection in second choice, which was similar to the national findings of BDHS [8]. Rural women were still not familiar with invasive procedures due to their poor literacy as well as lack of adequate FP information. Huda et al. also mentioned OCP as the first choice, followed by contraceptive injections after reviewing ten FP articles of Bangladesh related to contraceptive practices among married women of reproductive age [26]. In some places in Africa [27], after having a child long-acting reversible contraceptive (LARC) was found at the highest rate in contraceptive use. Use of condom as contraceptive was less than 5% in rural community that showed minimum male participation in fertility control and it was also matched with the findings of BDHS [8]. The probable reason of low percentage of male condom use might be the unwillingness of rural men to use it in male-dominant Muslim culture due to ignorance and misbelief. Rural men took the matter of MC use as only women’s responsibility. Huda et al. found slightly higher percentage of condom use (6.4%) which might be due to the inclusion of urban–rural respondents in that study [26].

Age, income, religion, family size, parity, etc. sometimes showed similarity with national findings of BDHS, 2014 [8] and in a few cases it might differ due to regional variation or due to the study process. Early marriage and adolescent pregnancy were common scenarios in rural community and these were almost similar with other studies on rural context of Bangladesh [8,9,22]. Preference for bigger family with more sons is very common in gender-stratified Bangladeshi rural culture [9,28]. It was not uncommon even in educated and economically stable families in rural areas [9,28].

SES, women’s empowerment, fertility preference and fertility control knowledge were taken as background issues of TPB model of fertility control behavior in this study. Although women’s age and family income were significant in the primary stage of hierarchical regression model, in the final stage these were not significantly related to fertility control behavior of rural women in this study that differed from many other studies on fertility control [9,22,23,26]. It might be due to the minimum variation in socioeconomic status of the respondents in rural community as poverty and inadequate literacy were a common scenario everywhere in rural Bangladesh. Another important reason might be putting them into background issues in TPB model focusing on the core components.

Women’s empowerment was in favor of fertility control behavior. Higher empowerment score showed better state in parity and contraceptive use. Importance of women’s empowerment in fertility control was also mentioned in other Bangladeshi papers [8,9]. Woldemicael [29] and Kamiya [30] also found significant association of women’s autonomy with reproductive behavior respectively in Eritrea and Tajikistan. Saleem and Pasha [31] described significance of women’s reproductive autonomy on contraceptive use in Pakistan. Although fertility preference was significantly associated with contraceptive use and fertility control in different studies [24,32], it did not show any significance in this study at the final stage of hierarchical regression model to predict fertility control behavior of Bangladeshi rural women. Jayraman et al. [32] found that fertility desire decreased and contraceptive use increased with increased number of children in South Asian countries like India, Nepal and Bangladesh. The findings of this study might differ due to the minimum variation in fertility preference of the respondents in rural community as son preference and preference for bigger family were common scenarios in rural Bangladesh. Fertility control knowledge showed significant effect on fertility control behavior of rural women which was similar to the findings of other FP papers [33,34]. Although fertility control knowledge was an important issue, a gap between knowledge and practice of contraceptives was found in many FP researches [35,36].

All the core factors of contraceptive use explained by TPB, except external control, showed significant effects on fertility control behavior of Bangladeshi rural women. Better FP attitude, positive social influence, higher PBC were in favor of better fertility control behavior of rural women that were described similarly in some studies using TPB [2,21]. High intention is very essential to get the planned behavior. Like several studies [2,21,37,38], it was found that high fertility control intention had a significant effect on contraceptive use. Although spousal status had a significant role on contraceptive use in different studies [39,40,41], it did not show any significance in this study. It might be due to the minimum variation in spousal relation among respondents in rural community of Bangladesh as male dominancy was very common and men were usually not interested in discussing fertility control matters. Regional variations were not only a significant issue in fertility control behavior among rural women in Bangladesh, they showed significance even in variations at predictor’s level. Ajzen and Klobas [21] also described regional variations of predictors in fertility decision-making across the European countries using TPB. Chittagong was found in a behind state, not only in women’s fertility control behavior but also in describing its determinants, which could explain the cause of regional variations in fertility control that was also a major concern in BDHS, 2014 [8]. Not only reducing the disparity in FP care access, women’s empowerment and health education on fertility issues should be more emphasized in family planning policies to minimize the regional disparity in fertility control.

## 5. Limitations

Respondents were randomly selected only from 3 divisions not covering all divisions to describe regional variations of fertility control behavior of rural women. Multistage stratified sampling based on national TFR at first stage minimized the selection bias and made the sample representative including respondents from all age groups. Rural women might be more positive in a few cases towards the items related to individual issues in MC use. As it was a cross-sectional survey, the study had few inherent limitations to describe the significance of predictors with fertility control behavior of women. Except these, the study adopted TPB model nicely to explain regional variations of fertility control behavior of rural women and its predictors. A mixed-method or qualitative study can be effective to explain more the reasons of regional disparity in MC use in rural Bangladesh based on current study findings.

## 6. Conclusions

Marked regional variations were observed in fertility control behavior of rural women along with its predictors. Regional disparity was an extra burden in male-dominant rural culture to achieve the national birth control target. Regional disparity should be minimized in contraceptive decision-making of rural women to ensure rational use of contraceptives. Fertility control awareness as well as income-generating programs for rural women could be helpful to improve their reproductive autonomy in fertility decision. The findings of this research will be helpful for planners to make current fertility control programs more region specific. Appropriate strategies should be included in the national fertility control program based on regional disparity in fertility indicators to make it more effective, not only to improve fertility control behavior of rural women but also to establish regional equity in fertility control.

## Figures and Tables

**Table 1:** Variables of hierarchical model to predict fertility control behavior of rural women.

Block 1	Block 2	Block 3	Outcome
Background Factors	Components from TPB	Regional Effect	
Age Educational level Monthly family income Number of children Women’s empowerment Fertility preference Fertility control knowledge	FP attitude Social influence PBC Fertility control intention Spousal status (actual behavioral control)	Region	Fertility control behavior

TPB = Theory of Planned Behavior, FP = Family planning, PBC = Perceived behavioral control.

**Table 2:** Socio-demographic characteristics of respondents (*n* = 1285).

Socio-Demographic Characteristics	Number	Percentage
**Age of respondents (years)**		
≤20	358	27.9
21–35	572	44.5
≥36	355	27.6
Mean (SD)	27.7 (±7.8) years	
Min–Max	18–46 years	
**Age of husbands (years)**		
≤25	151	11.8
26–35	672	52.2
≥36	462	36.0
Mean (SD)	34.8 (±8.7) years	
Min–Max	21–60 years	
**Religion**		
Islam	1153	89.7
Hindu	132	10.3
**Educational level of respondents**		
Illiterate	281	21.9
Primary	628	48.9
Junior School Certificate	275	21.4
Secondary School Certificate and above	101	7.8
**Educational level of husbands**		
Illiterate	343	26.7
Primary	529	41.2
Junior School Certificate	219	17.0
Secondary School Certificate and above	194	15.1
**Family income (Taka)**		
≤8000	489	38.1
8001–12,000	602	46.8
≥12,001	194	15.1
Mean (SD)	10,480 (±5261) Taka	
Min–Max	5000–60,000 Taka	
**Occupational status of respondents**		
Housewife	1152	89.6
Day laborer	66	5.1
Factory worker	29	2.3
Business	16	1.3
Govt./private employee	22	1.7
**Occupational status of husbands**		
Agricultural worker	710	55.2
Day laborer	223	17.4
Factory worker	89	6.9
Business	181	14.1
Govt./private employee	82	6.4
**Family type**		
Nuclear	478	37.2
Extended	807	62.8
**Number of family members**		
≤4	326	25.3
5–6	646	50.3
≥7	313	24.4
Mean (SD)	5.5 (±1.6)	
Min–Max	3–11	

**Table 3:** Reproductive characteristics of respondents (*n* = 1285).

Reproductive Characteristics	Number	Percentage
**Age at 1st marriage of respondents (years)**		
≤15	223	17.4
16–17	790	61.5
≥18	272	21.1
Mean (SD)	16.6 (±1.1) years	
Min–Max	14–20 years	
**Age at 1st pregnancy of respondents (years)**		
≤17	357	27.8
18–19	652	50.8
≥20	276	21.4
Mean (SD)	18.2 (±1.3) years	
Min–Max	16–22 years	
**Number of pregnancies**		
1	422	32.9
2	309	24.0
3–4	389	30.3
>4	165	12.8
Mean (SD)	2.5 (±1.5)	
Min–Max	1–8	
**Number of deliveries**		
1	425	33.1
2	316	24.6
3–4	423	32.9
>4	121	9.4
Mean (SD)	2.5 (±1.3)	
Min–Max	1–8	
**Number of living children**		
1	433	33.7
2	323	25.1
3–4	457	35.6
>4	72	5.6
Mean (SD)	2.4 (±1.3)	
Min–Max	1–7	
**Initial gap before 1st pregnancy (Years)**		
<2	618	48.1
≥2	667	51.9
Mean (SD)	1.6 (±0.7) years	
Min–Max	1–4 years	
**Use of contraceptives**		
Never	515	40.1
Sometimes	112	8.7
Very often	34	2.6
Always	624	48.6
**Types of modern methods (*n* = 770)**		
Oral pill	417	54.2
Injection	173	22.5
Norplant	28	3.6
Copper-T	21	2.7
Condom	58	**7.5**
Vasectomy/ligation	73	9.5

**Table 4:** Summary of hierarchical regression analysis for variables predicting rural women’s fertility control behavior (*n* = 1285).

Variable	Model1			Model2			Model3		
	*B*	*SE (B)*	*β*	*B*	*SE (B)*	*β*	*B*	*SE (B)*	*β*
Constant	−13.66	0.78		−20.00	0.88		−19.77	0.88	
Age	0.10	0.06	0.17 ***	0.06	0.02	0.10 *	0.04	0.02	0.07
Family income	3.8 × 10^−5^	0.00	0.04 *	2.2 × 10^−5^	0.00	0.03	1.8 × 10^−5^	0.20	0.02
Educational level									
Up to primary	1.33	0.30	0.15	0.71	0.27	0.08	0.61	0.27	0.07
Above primary	1.49	0.37	0.15	0.44	0.33	0.04	0.26	0.33	0.03
Number of children	0.02	0.15	0.01	0.12	0.13	0.04	0.19	0.13	0.05
Empowerment	0.20	0.02	0.23 ***	0.08	0.02	0.09 ***	0.08	0.02	0.09 ***
Fertility preference	0.28	0.03	0.21 ***	0.03	0.03	0.03	0.04	0.03	0.03
Fertility knowledge	1.02	0.06	0.39	0.53	0.06	0.20 ***	0.54	0.06	0.20 ***
FP attitude				0.17	0.03	0.12 ***	0.17	0.03	0.12 ***
Social influence				0.16	0.03	0.12 ***	0.15	0.03	0.11 ***
PBC				0.23	0.03	0.22 ***	0.23	0.03	0.22 ***
Spousal status				0.02	0.03	0.02	0.02	0.03	0.01
Fertility control intention				0.25	0.03	0.20 ***	0.25	0.03	0.20 ***
Regional status									
Dhaka division							0.46	0.19	0.05 *
Rajshahi division							0.51	0.20	0.05 *
*R* ^2^	0.556			0.664			0.666		
*F* for *R*^2^ change	199.49 ***			81.98 ***			4.06 *		

^(†)^ Educational level was represented as two dummy variables with illiterate serving as the reference group. In the case of regional status, Chittagong division was the reference group. One star (*) for just significant (*p* < 0.05) and three stars (***) for very highly significant (*p* < 0.001).

**Table 5:** Regional variations in fertility control behavior and its related factors by Kruskal–Wallis and one-way ANOVA test (*n* = 1285).

Characteristics	Mean Score ± (SD)				*p*-Value
	National (*n* = 1285)	Rajshahi (*n* = 426)	Dhaka (*n* = 429)	Chittagong (*n* = 430)	
Fertility control behavior	8.9 (4.6)	9.7 (4.5)	9.1 (4.7)	7.8 (4.3)	<0.001 ***
FP attitude	30.1 (3.3)	30.6 (3.3)	30.1 (3.2)	29.5 (3.4)	<0.001 ***
Social influence	29.0 (3.6)	29.6 (3.3)	29.3 (3.5)	28.0 (3.4)	<0.001 ***
PBC	21.6 (4.3)	22.2 (4.1)	21.6 (4.3)	20.9 (4.5)	<0.001 ***
Fertility control intention	20.9 (3.6)	21.5 (3.2)	20.8 (3.4)	20.2 (4.0)	<0.001 ***
Spousal status	7.6 (3.1)	8.0 (3.1)	7.8 (3.2)	6.9 (2.8)	<0.001 ***
Fertility preference	20.1 (3.5)	20.5 (3.4)	20.1 (3.4)	19.7 (3.6)	<0.01 **
Fertility control knowledge	5.6 (1.7)	5.8 (1.7)	5.6 (1.6)	5.5 (1.8)	0.120
Women’s empowerment	34.3 (5.2)	35.7 (5.0)	34.1 (5.4)	33.1 (4.8)	<0.001 ***

Two stars (**) for highly significant (*p* < 0.01) and three stars (***) for very highly significant (*p* < 0.001).
